# Evaluation and Management of Dyspnea in Hypermobile Ehlers-Danlos Syndrome and Generalized Hypermobility Spectrum Disorder: Protocol for a Pilot and Feasibility Randomized Controlled Trial

**DOI:** 10.2196/44832

**Published:** 2023-03-20

**Authors:** Dmitry Rozenberg, Noor Al Kaabi, Encarna Camacho Perez, Sahar Nourouzpour, Laura Lopez-Hernandez, Laura McGillis, Ewan Goligher, W Darlene Reid, Chung-Wai Chow, Clodagh M Ryan, Dinesh Kumbhare, Ella Huszti, Kateri Champagne, Satish Raj, Susanna Mak, Daniel Santa Mina, Hance Clarke, Nimish Mittal

**Affiliations:** 1 Respirology and Lung Transplantation Toronto General Hospital Research Institute University Health Network Toronto, ON Canada; 2 Temerty Faculty of Medicine University of Toronto Toronto, ON Canada; 3 GoodHope Ehlers-Danlos Syndrome Clinic University Health Network Toronto, ON Canada; 4 Respirology Toronto General Hospital Research Institute University Health Network Toronto, ON Canada; 5 Interdepartmental Division of Critical Care Medicine University of Toronto Toronto, ON Canada; 6 KITE—Toronto Rehab University Health Network Toronto, ON Canada; 7 Physical Therapy University of Toronto Toronto, ON Canada; 8 Faculty of Kinesiology and Physical Education University of Toronto Toronto, ON Canada; 9 Biostatistics Research Unit University Health Network Toronto, ON Canada; 10 Institut de Médecine du Sommeil Montreal, QC Canada; 11 Department of Cardiac Sciences Cumming School of Medicine Calgary, AB Canada; 12 Department of Cardiology Mount Sinai Hospital Toronto, ON Canada; 13 Department of Anesthesia and Pain Management University Health Network Toronto, ON Canada

**Keywords:** Ehlers-Danlos Syndrome, generalized hypermobility spectrum disorders, inspiratory muscle training, rehabilitation, exercise, mobile phone

## Abstract

**Background:**

Dyspnea is a prevalent symptom in individuals with hypermobile Ehlers-Danlos Syndrome (hEDS) and generalized hypermobility spectrum disorder (G-HSD), yet its contributors have not been identified. One known contributor to dyspnea is respiratory muscle weakness. The feasibility and effectiveness of inspiratory muscle training (IMT) in combination with standard-of-care rehabilitation (aerobic, resistance, neuromuscular stabilization, and balance and proprioception exercises) in improving respiratory muscle strength and patient-reported outcomes in patients with hEDS or G-HSD have not been evaluated.

**Objective:**

This study aims to evaluate dyspnea, respiratory muscle strength, and patient-reported outcome measures (PROMs) in hEDS or G-HSD compared with healthy controls and to assess the feasibility of a randomized controlled trial of IMT and standard-of-care rehabilitation for improving respiratory muscle strength, exercise capacity, and PROMs compared with standard-of-care rehabilitation in hEDS and G-HSD.

**Methods:**

The study will include 34 participants with hEDS or G-HSD and 17 healthy, age- and sex-matched controls to compare respiratory muscle structure and function and PROMs. After baseline assessments, participants with hEDS or G-HSD will be randomized into the intervention group and provided IMT combined with Ehlers-Danlos Syndrome standard-of-care rehabilitation or into the usual care group, and provided only standard-of-care rehabilitation for 8 weeks. The intervention group will be prescribed IMT in their home environment using the POWERbreathe K5 IMT device (POWERbreathe International Ltd). IMT will comprise 2 daily sessions of 30 breaths for 5 days per week, with IMT progressing from 20% to 60% of the baseline maximal inspiratory pressure (MIP) over an 8-week period. Feasibility will be assessed through rates of recruitment, attrition, adherence, adverse events, and participant satisfaction. The primary pilot outcome is MIP change over an 8-week period in hEDS or G-HSD. Secondary outcomes will include the evaluation of dyspnea using Medical Research Council Scale and 18-point qualitative dyspnea descriptors; diaphragmatic thickening fraction using ultrasound; respiratory muscle endurance; pulmonary function; prefrontal cortical activity using functional near-infrared spectroscopy; aerobic capacity during cardiopulmonary exercise testing; quality of life using Short Form-36; and scores from the Depression, Anxiety, and Stress scale-21. These measures will also be performed once in healthy controls to compare normative values. Multivariable regression will be used to assess the contributors to dyspnea. Paired 2-tailed *t* tests will be used to assess the changes in MIP and secondary measures after 8 weeks of IMT.

**Results:**

Study recruitment began in August 2021 and, with several disruptions owing to COVID-19, is expected to be completed by December 2023.

**Conclusions:**

This study will provide a better understanding of the factors associated with dyspnea and the feasibility and effectiveness of IMT combined with standard-of-care rehabilitation. IMT may be a novel therapeutic strategy for improving respiratory muscle function and patient-reported outcomes in individuals with hEDS or G-HSD.

**Trial Registration:**

ClinicalTrials.gov NCT04972565; https://clinicaltrials.gov/ct2/show/NCT04972565

**International Registered Report Identifier (IRRID):**

DERR1-10.2196/44832

## Introduction

### Background

Ehlers-Danlos Syndrome (EDS) is a group of hereditary connective tissue disorders characterized by multisystem manifestations involving musculoskeletal and nonmusculoskeletal domains [1]. Currently, the International Classification of EDS and related disorders includes 14 subtypes based on a set of clinical criteria, with the most common subtype being hypermobile EDS (hEDS), which comprises 80% to 90% of EDS cases [1-3]. Patients who present with symptomatic generalized joint hypermobility and chronic pain but do not meet the International 2017 classification criteria are classified as having generalized hypermobility spectrum disorder (G-HSD) [3]. The clinical manifestations of the disease vary according to the EDS subtype, and our understanding of this disorder has evolved to recognize multisystem manifestations beyond the musculoskeletal system, including gastrointestinal, neurological, cardiovascular, and respiratory involvement [1]. These manifestations arise from abnormal collagen or proteins that bind collagen that affect the connective tissue throughout the body [1]. Given the multisystem nature of hEDS and G-HSD, several symptoms such as pain and fatigue may contribute to reduced health-related quality of life (HRQL), physical deconditioning, anxiety, and depression [4-8].

### Respiratory Manifestations in EDS and G-HSD

Our research group and others have recently highlighted that respiratory manifestations are common in hEDS and G-HSD and include, but are not limited to, dyspnea, cough, asthma, and respiratory muscle weakness [2,9]. The collagen abnormalities lead to impairments throughout the respiratory system, which may contribute to the respiratory abnormalities observed in this population [2,10]. In individuals with hEDS, the prevalence of dyspnea is estimated to be approximately 50% [11]. Dyspnea has been described in a few studies on individuals with G-HSD, but its prevalence and determinants have not been well characterized [12]. A study reported that up to 26% of the patients with G-HSD presented with cardiorespiratory manifestations such as dyspnea, palpitations, and chest pain, which were commonly attributed to autonomic dysfunction [13].

Respiratory symptoms in EDS and G-HSD have also been associated with worse outcomes such as lower physical activity levels and function. Individuals with EDS or G-HSD experienced increased dyspnea while walking on a flat level compared with healthy controls [12]. Furthermore, a study investigating physical activity levels between patients with hEDS or G-HSD with autonomic dysfunction and those without autonomic dysfunction observed that autonomic dysfunction was associated with exercise intolerance, which is often attributed to increased symptoms of dyspnea and chest pain [14]. Postural orthostatic tachycardia syndrome, a more severe form of autonomic dysfunction, has also been observed to be associated with dyspnea, chest pain, and impairments in HRQL [15,16]. Thus, respiratory manifestations in individuals with hEDS or G-HSD have important consequences on exercise tolerance, HRQL, and functional abilities.

### Contributors to Dyspnea in hEDS and G-HSD

Functional impairments in respiratory muscle weakness occur in >75% of individuals with hEDS and can result in dyspnea and a restrictive pattern of ventilation [11]. The diaphragm is the main respiratory muscle, and alterations in collagen can lead to impairments in the respiratory muscle structure and function [17]. Respiratory muscle weakness has also been associated with lower vital capacity, which may contribute to dyspnea [18,19]. Thus, strengthening the respiratory muscles through modalities such as inspiratory muscle training (IMT) may improve dyspnea and pulmonary function [20].

Some individuals with hEDS or G-HSD have been observed to have chest wall deformities and increased rates of asthmatic symptoms, which can contribute to dyspnea and peripheral airway abnormalities [12,21,22]. There is also an increased tendency for dynamic airway collapse despite normal chest imaging, which highlights altered lung mechanics, including airway pressures and lung volumes [12]. Furthermore, deconditioning has been described as a contributor to low physical activity levels in the population with EDS or G-HSD as a consequence of increased joint laxity, pain, and peripheral muscle weakness [23]. Physical deconditioning may further exacerbate dyspnea and contribute to low exercise tolerance [23]. In conditions such as chronic obstructive pulmonary disease (COPD), cortical neural activity in the prefrontal cortex (PFC) has been shown to be associated with dyspnea and exercise intolerance and may be another contributor to dyspnea in hEDS or G-HSD [24,25]. Modalities such as functional near-infrared spectroscopy (fNIRS) have been used to assess PFC activity in individuals experiencing dyspnea during exercise testing [24]. Taken together, respiratory muscle weakness, peripheral airway changes, physical deconditioning, and autonomic dysfunction may contribute to the multifactorial nature of dyspnea in hEDS or G-HSD. These factors can be evaluated clinically and may help us understand the mechanisms that contribute to dyspnea and, importantly, tailor effective respiratory management strategies.

### IMT and Standard-of-Care Rehabilitation

IMT has been established as an effective intervention for improving respiratory muscle structure and function in populations with chronic lung diseases [26,27]. A study observed that among 104 individuals with hEDS, 80 (77%) individuals had respiratory muscle weakness [11]. After 6 weeks of IMT, respiratory muscle strength measured with sniff nasal inspiratory pressure improved by 20% (pre-IMT intervention: mean 41, SD 17 cm H_2_O vs post-IMT measurement: mean 49, SD 18 cm H_2_O; *P*<.001) in patients with hEDS. Furthermore, the 6-minute walk distance improved by 13% (pre-IMT measurement: mean 455, SD 107 m vs post-IMT measurement: mean 515, SD 127 m; *P*=.003) along with forced expiratory volume in 1 second by 10% (pre-IMT measurement: mean 94%, SD 14% vs post-IMT measurement: mean 103%, SD 11%; *P*=.01). Thus, IMT may be an effective intervention for improving respiratory muscle strength, lung function, and exercise capacity in patients with hEDS or G-HSD.

Physical rehabilitation involving whole-body exercises such as aerobic and strength training has been shown to improve dyspnea in healthy populations and populations with chronic lung diseases such as COPD and idiopathic pulmonary fibrosis [28-31]. In a meta-analysis of 14 randomized controlled trials (RCTs), resistance training and endurance training were associated with improved dyspnea tolerance during exercise training, along with resting dyspnea levels, in patients with COPD [30]. This is often attributed to improved physical fitness owing to exercise training, which increases the efficiency of the muscles and decreases the level of ventilatory demand [30]. Unlike other populations, research on the effects of exercise training on individuals with hEDS or G-HSD is in its early stages, with only 5 RCTs performed in this area to date [32]. Following rehabilitation, in patients with hEDS or G-HSD, improvements have been observed in exercise capacity, muscle strength, mood, HRQL, and performance of daily activities [32].

Although the IMT RCT highlighted the safety and feasibility of IMT in hEDS, the study did not evaluate the mechanisms of dyspnea or assess diaphragm structure and function [11]. Furthermore, physical activity and other concurrent training, which may provide synergistic beneficial effects on respiratory symptoms and exercise capacity, were not considered. Several studies on chronic lung disease have demonstrated that IMT in combination with whole-body exercises, such as aerobic exercise, offers synergistic benefits on respiratory muscle strength and endurance and exercise capacity in comparison with whole-body exercises alone [23,33,34]. IMT alone has been shown to be an effective intervention in populations with asthma, populations with COPD, and populations with interstitial lung disease for improving maximal inspiratory pressure (MIP), respiratory muscle endurance, and diaphragm function [26,27,35] and thus may be an effective strategy in the population with hEDS or G-HSD.

The efficacy of both IMT and standard-of-care rehabilitation in ameliorating dyspnea and improving aerobic capacity and respiratory muscle structure and function in the population with hEDS or G-HSD has not been investigated to date. It is also unclear whether IMT in combination with standard-of-care rehabilitation is a feasible intervention, specifically for the population with hEDS or G-HSD, given the increased comorbidities. Furthermore, the efficacy of IMT has only been evaluated in hEDS and yet to be studied in G-HSD. This study aims to address these gaps.

### Study Aims

The aims of this study are (1) to compare respiratory muscle strength and patient-reported outcome measures (PROMs) between individuals with hEDS or G-HSD and healthy, age- and sex-matched controls, as well as evaluate the main contributors to dyspnea in individuals with hEDS or G-HSD, and (2) to assess the feasibility of an RCT of IMT and standard-of-care rehabilitation (aerobic, resistance, neuromuscular stabilization, and balance and proprioception exercises) for improving respiratory muscle strength, exercise capacity, and PROMs compared with standard-of-care rehabilitation in hEDS or G-HSD.

### Hypotheses

We hypothesize that (1) individuals with hEDS or G-HSD will have lower respiratory muscle strength, higher peripheral airway resistance, lower HRQL, and higher anxiety and depression levels than age- and sex-matched healthy controls and that (2) the delivery of IMT and standard-of-care rehabilitation will be feasible and result in a significant improvement in respiratory muscle strength (MIP) over an 8-week period.

## Methods

### Study Design

Participants with hEDS or G-HSD will complete assessments at baseline (T_0_; Table 1) and will be randomized into the intervention arm (IMT and standard-of-care rehabilitation) or usual care group (standard-of-care rehabilitation). The standard-of-care rehabilitation at the GoodHope EDS Clinic at Toronto General Hospital comprises active exercise training involving aerobic, resistance, neuromuscular stabilization, and balance and proprioception exercises, and is a part of the GoodHope Exercise and Rehabilitation Program (GEAR) [36].

Age- and sex-matched healthy controls will undergo the same T_0_ assessments as those that the EDS and G-HSD intervention and usual care groups will undergo. The respiratory muscle structure and function, dyspnea, exercise capacity, and collected PROMs will be compared. Only the participants with hEDS or G-HSD (intervention and usual care groups) will repeat the T_0_ assessments after 8 weeks, as described in the subsequent section on assessments.

**Table 1:** Summary of study measurements.

Measurement		Baseline assessments^a^	
		Intervention and usual care groups	Healthy control group
**Dyspnea**			
	Medical Research Council Dyspnea Scale	✓	✓
	18-Point Qualitative Dyspnea Scale	✓	✓
**Pulmonary function tests**			
	Oscillometry	✓	✓
	Spirometry and lung volumes	✓	✓
	Respiratory muscle endurance^b^	✓	✓
	Maximal respiratory pressures	✓	✓
	Diaphragmatic measures	✓	✓
**PROMs^c^**			
	Health-related quality of life: Short Form-36	✓	✓
	Mood: Depression, Anxiety, and Stress scale	✓	✓
	Physical activity volume: Godin Leisure-Time Exercise Questionnaire	✓	✓
	Demographic questionnaire	✓	✓

^a^All baseline measures will be repeated after 8 weeks for the participants with hypermobile Ehlers-Danlos Syndrome or generalized hypermobility spectrum disorder.

^b^Respiratory muscle endurance will be repeated at 4 weeks.

^c^PROM: patient-reported outcome measure.

### Ethics Approval

Ethics approval has been obtained from the research ethics board at the University Health Network (ID: 20-6346.0) and University of Toronto (ID: 42667). The study has been registered at ClinicalTrial.gov (NCT04972565).

**Figure 1:**
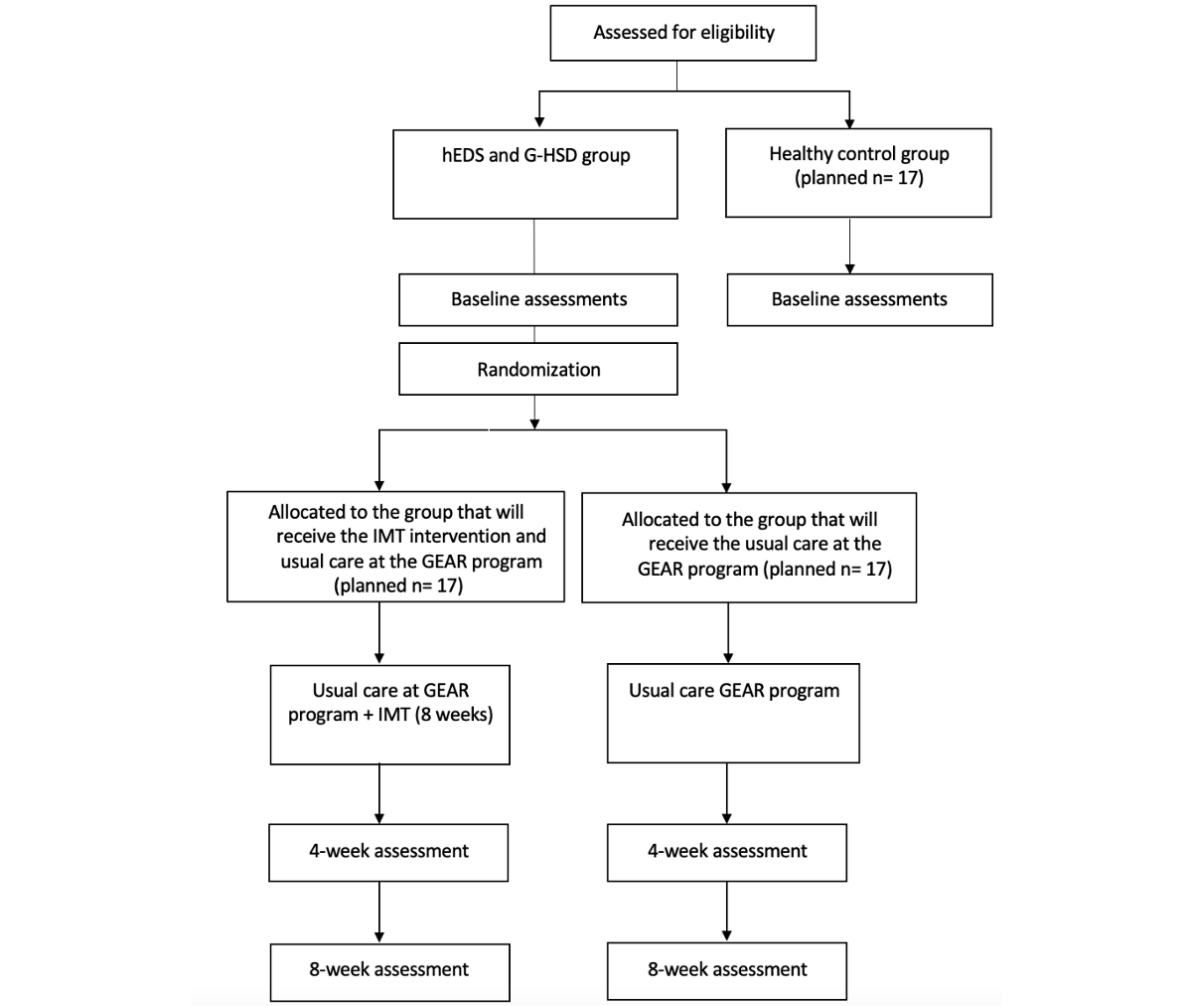
CONSORT (Consolidated Standards of Reporting Trials) flow diagram with healthy controls. GEAR: GoodHope Exercise and Rehabilitation Program; G-HSD: generalized hypermobility spectrum disorder; hEDS: hypermobile Ehlers-Danlos Syndrome; IMT: inspiratory muscle training.

Written informed consent will be obtained from all participants before the commencement of any research activities. The Standard Protocol Items: Recommendations for Interventional Trials, the CONSORT (Consolidated Standards of Reporting Trials) statement, and the Consensus on Exercise Reporting Template will be used [37-39]. Refer to Figure 1 for the CONSORT flow diagram.

### Recruitment and Screening

Participants with hEDS or G-HSD will be recruited from the GEAR program at Toronto General Hospital, which is a major teaching hospital in Toronto, Canada. The GEAR program evaluates approximately 12 new participants per month. Potential participants are screened for study eligibility by exercise professionals who are part of the GEAR program. The research team will confirm the eligibility criteria, discuss study participation, and obtain consent among interested participants. The study flow of participants is shown in Figure 1.

### Participants With hEDS or G-HSD

The sample will comprise adult individuals (aged ≥18 years) diagnosed with hEDS or G-HSD by the EDS medical team at Toronto General Hospital based on the 2017 diagnostic criteria [3]. Participants will be included if they have started their participation in the GEAR program within 4 weeks.

The following exclusion criteria will be applied:

1. Diagnosis of other subtypes of EDS (eg, classical and vascular)
2. Have a cardiac pacemaker, defibrillator, neuromuscular disease, severe autonomic dysfunction, or other cardiovascular manifestations limiting exercise or other exercise contraindications
3. Recent respiratory infection (<1 month) or known diagnosis of obstructive or restrictive parenchymal disease
4. History of pneumothorax, acute otitis media (fluid behind the eardrum), or rupture of tympanic membranes, given the risk owing to IMT
5. Participation in formal exercise training or IMT program within the last 3 months
6. Insufficient English fluency to provide informed consent or inability to follow study protocols
7. Self-reported pregnancy
8. No internet access for IMT or web-based assessments

### Healthy Controls

Healthy adults (aged ≥18 years) will be age- and sex-matched with the participants with EDS and G-HSD. Individuals will be excluded from the study if they meet the following criteria:

1. Diagnosis of a respiratory, cardiovascular, or musculoskeletal condition that may contribute to exercise limitation
2. Presence of cardiac pacemaker or implantable defibrillator
3. Insufficient English fluency to provide informed consent or inability to follow study protocols
4. Self-reported pregnancy
5. Active smoker (cigarettes or marijuana)

### Assessments

At T_0_, the participants with hEDS and G-HSD will undergo pulmonary function tests (PFTs), cardiopulmonary exercise test (CPET) with fNIRS on the PFC, PROMs, and diaphragm ultrasound. The same assessments will be performed for healthy controls. Please refer to Table 1 for a detailed list of T_0_ measurements. At 4 weeks, respiratory muscle endurance assessment will be repeated for the participants with hEDS and G-HSD. All T_0_ assessments, including PFTs, CPET, PROMs, and respiratory muscle endurance assessment, will be repeated at 8 weeks for the participants with hEDS and G-HSD.

The following tests will be used to assess the primary and secondary outcome measures in the PFT laboratory at Toronto General Hospital.

### Respiratory Parameters

#### Assessment Using PFTs

##### Spirometry and Lung Volumes

Forced expiratory volume in 1 second, forced vital capacity, total lung capacity, and lung volumes (tidal volume, inspiratory capacity, expiratory reserve, and residual volumes) will be evaluated in accordance with the American Thoracic Society guidelines before CPET [40].

##### Oscillometry

This will be performed before other PFTs according to the European Respiratory Society Guidelines, as previously described [41,42]. Oscillometry provides detailed information regarding respiratory mechanics by measuring respiratory impedance, a complex sum of respiratory resistance, and reactance [43]. This technique uses sound waves to capture disturbances in airway resistance and can be a more sensitive marker of peripheral airway obstruction than spirometry [44,45]. Because it is performed during normal breathing, oscillometry is independent of effort and is not influenced by respiratory muscle strength. We will follow the quality assurance and control guidelines developed by our group using the tremoflo device (Thorasys) [46].

##### Respiratory Muscle Strength

MIP and maximal expiratory pressure will be evaluated using the American Thoracic Society standards. These measures will be repeated 3 times, and the highest value (cm H_2_O) that is 10% within the lower measures will be used [47,48].

##### Respiratory Muscle Endurance

The threshold loading device (Threshold IMT trainer [Philips]) [49] will be used to evaluate respiratory muscle endurance. We will assess the time that can be sustained at respiratory endurance at 30% of MIP obtained as part of the T_0_ measures after a brief familiarization session with the loading device. The maximum endurance that the threshold loading device can reach is 41 cm H_2_O. The endurance assessment will be repeated within 5 minutes if the participant stops the protocol early (<1 minute) because of severe coughing or lack of familiarity with the loading device. The maximum endurance that participants will be asked to perform is 15 minutes. Oxygen saturation will be concurrently monitored with pulse oximetry during the assessment.

##### Diaphragm Ultrasound

While the participant is in semirecumbent position, the research coordinator will place an ultrasound probe perpendicular to the chest wall in the 8th or 9th intercostal space between the anterior and midaxillary lines using a 5-13 MHz transducer (GE Logiq e Laptop Ultrasound System [GE Medical Systems]) [50]. Bright-mode ultrasound will be used to visualize the diaphragm, and motion-mode ultrasound will be used to assess the amplitude of the cranio-caudal diaphragmatic excursion during quiet and deep breathing. Diaphragm thickness will be measured as the distance between the leading edges of the diaphragmatic pleura and peritoneum at resting end expiration and after peak inspiration. Thickening fraction, an established marker of diaphragm strength, will be calculated as follows: ([diaphragm thickness at end expiration–diaphragm thickness at peak inspiration] divided by diaphragm thickness at end expiation) × 100 [50].

#### Assessment Using CPET

##### Overview

Symptom-limited CPET will be performed on a cycle ergometer (Elema), with exhaled gas measurements captured breath by breath, as routinely applied in our PFT laboratory. Testing will start with 2 minutes of complete rest on the bike, followed by unloaded pedaling for 2 minutes, and then exercise intensity will be increased by 10 to 20 W per minute using a ramp protocol. Blood pressure, 12-lead electrocardiogram, and peripheral oxygen saturation will be continuously monitored. Inspiratory capacity, tidal volumes, and respiratory rate will be measured throughout the exercise to assess for any dynamic hyperinflation and ventilatory reserve. Heart rate (HR) response, peak aerobic capacity, oxygen pulse (measure of cardiac stroke volume), and minute ventilation (product of tidal volume and respiratory rate) will be collected throughout the exercise and up to 2 minutes after test completion with unloaded pedaling, with percent predicted values reported. HR recovery (difference between peak HR and HR 1 minute after exercise) and the chronotropic response index ([peak HR – resting HR × 100] / [220 – age] – [resting HR]) will be collected as a measure of autonomic function [51-53]. Chronotropic incompetence is the inability to decrease HR after exercise or the inability to reach 80% of the maximal age-predicted HR because of abnormal sympathetic activation [51]. Symptom intensity for breathing and leg discomfort with exercise will be measured at rest and every 3 minutes throughout the exercise using the Borg dyspnea [54] and leg fatigue scales [55]. The reasons for exercise cessation will be ascertained from the participants (ie, dyspnea, leg fatigue, both, or other). To ensure participant safety before CPET, 12-lead electrocardiograms will be performed for both the participants with hEDS or G-HSD and healthy controls.

##### Assessment Using fNIRS

fNIRS is a noninvasive method that uses continuous optical topography to evaluate increases in oxygenated hemoglobin levels, an indicator of PFC activity that has been suggested to be associated with dyspnea [56-58]. The change in oxyhemoglobin and deoxyhemoglobin will be monitored using the Artinis fNIRS device (Artinis Medical Systems) [59], which will be secured over the participant’s forehead, with the center of each sensor pad vertically aligned with the iris. Light intensity data at 2 wavelengths (730 and 850 nm) will be obtained during CPET from the left and right sides of medial PFC and dorsolateral PFC using a cognitive optical brain imaging software at a sampling frequency of 4 Hz. Detector gain settings will be adjusted for light intensity, and filters will be applied to attenuate the physiological artifacts of respiration and pulse.

#### Assessment Using PROMs

##### Dyspnea

The Medical Research Council (MRC) dyspnea scale [60], Borg dyspnea scale [54], and 18-item qualitative dyspnea descriptors will be used [61]. This will allow us to evaluate the quantitative and qualitative changes in respiratory symptoms.

1. *MRC dyspnea scale*: the MRC dyspnea scale (1-5) will be used to assess the effect of breathlessness on daily activities at T_0_ [60]. It allows patients to indicate the extent to which breathlessness limits their daily functioning [60].
2. *Borg dyspnea scale*: the highest score on a 10-point Borg scale at the beginning and end of CPET will be measured. The participants will also be asked to indicate their daily Borg dyspnea score before and after their IMT practice in their participant logs [62].
3. *Qualitative dyspnea descriptors:* the qualitative dyspnea descriptors scale comprises a list of 18 dyspnea descriptors. Qualitative descriptors of dyspnea will be ascertained at T_0_ and at the end of CPET [61].

##### Exercise Behaviors and Physical Activity

Exercise behaviors will be measured using the 3-item Godin Leisure-Time Exercise Questionnaire—Leisure Score Index, which is a reliable and validated measure for this population [63]. This index assesses the frequency of mild, moderate, and strenuous bouts of leisure physical activity performed for at least 15 minutes over the past week. In addition, physical activity will be measured using a tracking device (Fitbit), which will record daily steps and active minutes for the study participants with hEDS or G-HSD [64,65].

##### Measurement of HRQL

The Short Form-36 Health Survey (version 1.0) will be used to assess generic HRQL [66]. It comprises 8 health domains that contribute to 2 physical and mental component scores (ranging from 0 to 100), with higher scores representing better HRQL. A 5-point change in the physical component scores or mental component scores is considered clinically significant [66].

##### Depression, Anxiety, and Stress

The Depression, Anxiety, and Stress scale has 21 items to assess mood (depression, anxiety, and stress) and has been administered to patients with hEDS [8]. It has acceptable to excellent internal consistency, and normative data are available [67].

### Anthropometric Measures

Standard anthropometric measures will be captured for all the participants. Height, weight, waist circumference, and BMI information will be collected for the participants with hEDS or G-HSD from the electronic patient charts. The same measures will be collected for healthy controls during the T_0_ assessments.

### Clinical Assessments

We will abstract standard GEAR program measures from the medical charts for all the participants with hEDS or G-HSD: 6-minute walk distance, lower extremity function (5-repetition sit-to-stand test), hand-grip strength, Bristol questionnaire, and Godin Leisure-Time Exercise scale. The Beighton score [68], Charlson comorbidity index [69], and medications such as opioids that can impact dyspnea will be abstracted from chart review.

### Sociodemographic Parameters

We will abstract information on demographics such as age, ethnicity, sex, employment status, education level, and lifestyle factors (eg, smoking status) from the medical charts for the participants with hEDS or G-HSD. Demographic data will be collected from healthy controls via a demographic survey.

### Randomization

After T_0_ assessments, the participants will be randomly allocated to the intervention or usual care group using a 1:1 ratio stratified by hEDS or G-HSD. The allocation of the participants to their groups will be randomized (blocks of 2 to 4) using shuffled, opaque envelopes by a biostatistician. The study participants and the research team and coordinator supervising the IMT will be aware of the group assignments.

### Study Arms

#### Overview

The participants in both the intervention and usual care groups will receive the standard-of-care rehabilitation in the GEAR program. Those in the intervention group will receive IMT in addition to the GEAR program components, as shown in Table 2.

**Table 2:** Summary of the study procedures.

Study groups	IMT^a^ intervention group with participants with hEDS^b^ or G-HSD^c^ (n=17)	Usual care group with participants with hEDS or G-HSD (n=17)
Standard of care—GEAR^d^ program: baseline (T_0_), 4 weeks from T_0_ (T_1_), 8 weeks from T_0_ (T_2_), and 20 weeks from T_0_ (T_3_)	Aerobic, resistance, neuromuscular stabilization, and balance and proprioception exercises [36]	Aerobic, resistance, neuromuscular stabilization, and balance and proprioception exercises [36]
Baseline assessment (details provided in Table 1)	Baseline assessment: explanation of equipment and tracking IMT device POWERbreathe K5 IMT and exercise logs and Fitbit device and instructions Fitbit device and instructions Exercise logs	Baseline assessment: Fitbit device and instructions Exercise logs
Study intervention: 8 weeks	2 daily IMT sessions of 30 breaths (<5 minutes per session) for 5 days per week, up to 8 weeks IMT starting at 20% of MIP^e^ (up to a maximum of 60%) Intensity progressed by 5% to 10% if the median weekly Borg dyspnea score is <7 Web-based supervision by the study team Weekly check-in with the study coordinator	N/A^f^
Follow-up: week 4 time point	Study assessments will be performed at week 4 from the home environment. Exercise logs and respiratory muscle endurance will be assessed	Study assessments will be performed at week 4 from the home environment. Exercise logs and respiratory muscle endurance will be assessed
End point: week 8 time point	Study assessments conducted at baseline will be performed again at week 8 in the PFT^g^ laboratory. Collection of logs, Fitbits, and IMT device (POWERbreathe K5) will be done at the end of the study	Study assessments conducted at baseline will be performed at week 8 in the PFT laboratory. Collection of logs, Fitbits, and IMT device (POWERbreathe K5) will be done at the end of the study.

^a^IMT: inspiratory muscle training.

^b^hEDS: hypermobile Ehlers-Danlos Syndrome.

^c^G-HSD: generalized hypermobility spectrum disorder.

^d^GEAR: GoodHope Exercise and Rehabilitation Program.

^e^MIP: maximal inspiratory pressure.

^f^N/A: not applicable.

^g^PFT: pulmonary function test.

#### Usual Care Group

The standard-of-care rehabilitation in the GEAR program comprises 8 weeks of aerobic, resistance, neuromuscular stabilization, and balance and proprioception exercises performed in the home environment [1]. The participants in the GEAR program attend in-person or web-based exercise training sessions at the following intervals: T_0_, 4 weeks from T_0_, 8 weeks from T_0_, and 20 weeks from T_0_ to observe maintenance [46]. The GEAR program comprises three major elements: (1) an individualized home-based rehabilitation and exercise therapy, (2) education on the self-management of hEDS or G-HSD symptoms, and (3) a community resource engagement plan. These components are delivered by a team of physiotherapists and kinesiologists.

#### Intervention Group (IMT)

The participants in the intervention group will receive an individual and personalized prescription for an IMT program for 8 weeks. This IMT program will use the POWERbreathe K5 IMT device in the participants’ home environment combined with the standard-of-care rehabilitation as part of the GEAR program, as described earlier. A research coordinator will facilitate these training sessions and monitor the participants via the web for the first 3 sessions in the first week and once a week from weeks 2 to 8. Over 8 weeks, 2 daily IMT sessions of 30 breaths (<5 min/session) will be performed for 5 days per week, starting with 20% of the participants’ T_0_ MIP up to a maximum of 60%. This protocol was developed based on the previous literature on IMT applied in lung transplant candidates at our center [70-72]. The total workload per session (product of inspiratory muscle force and inhaled volume expressed in Joules) will be abstracted from the IMT device. Additional variables abstracted from the device will include inspiratory muscle load (cm H_2_O), mean power per breath (W), and mean volume per breath (Liters).

The participants will be asked to record their resting and post-IMT Borg dyspnea scores for 5 days of the week (2 sessions per day), along with any side effects for each session. The median Borg dyspnea score for the previous week will be reviewed by the research coordinator, and IMT intensity will be increased by 5% to 10% of the T_0_ MIP if the median weekly Borg dyspnea score is <7 (very severe breathlessness with IMT) [54].

The participants will be asked to maintain IMT diaries to assess adherence. They will receive instructions and feedback on how to optimize their home training efforts based on direct observation of their IMT with the POWERbreathe K5 device and review of IMT workload from the device. They will have the possibility to schedule additional sessions with the study team during the week, if further support is needed.

### Trial Outcomes

#### Estimates of Intervention Feasibility

Study feasibility will be assessed through recruitment rate, attrition, IMT program adherence, safety, and satisfaction (Textbox 1).

**Feasibility estimates.**

- Recruitment
  We will evaluate the recruitment rate as the number of participants who consented and enrolled relative to the total number of eligible patients approached for eligibility assessment by the research coordinator. The reasons for nonparticipation in the study will be documented. A successful recruitment rate will be defined as 20% in this study.
- Retention
  Retention will be evaluated through participant attrition throughout the intervention period. For each arm of the study, the retention rate will be assessed by measuring the number of participants who completed all assessments compared with the number of participants who enrolled and randomized. A successful retention rate will be defined as ≥80% in this study.
- Adherence
  Adherence to inspiratory muscle training (IMT) in the intervention group will be tracked electronically and through participant logs (number of completed sessions of 30 breaths out of 10 planned sessions per week) with the IMT device over the 8-week period. Adherence will also be assessed during weekly communications with participants as well as through completed daily IMT logs, which will capture IMT training days and IMT prescription (percentage of maximal inspiratory pressure). These data will also be verified after the IMT device is returned. The number of completed IMT sessions will be reviewed from the recordings on the device.
  The physical activity and exercise behaviors prescribed through the GoodHope Exercise and Rehabilitation Program will be monitored in both the intervention and usual care groups. Daily step counts will be assessed from the Fitbit device. The participants with hypermobile Ehlers-Danlos Syndrome or generalized hypermobility spectrum disorder will be asked to download the Fitbit app on their smartphones or tablets. A 1-page document highlighting the key steps for setting up the Fitbit tracker will be provided. We will also work with the participants via the web to set up the device if any problems arise. The exercise behaviors of the study participants in both groups will be reviewed using the self-reported exercise logs, which capture the exercise type, frequency, intensity, and duration on a daily basis. At the end of the baseline assessment, the study participants in both the usual care and intervention groups will be provided with documents explaining the maintenance and use of the study devices (eg, Fitbit, respiratory muscle endurance trainer, and IMT device for the intervention group).
- Safety
  The participants will log any adverse effects during the IMT program, which will be reviewed weekly by the research coordinator. Adverse effects are rare; however, symptoms previously observed during IMT include mild chest discomfort, worsening respiratory symptoms (eg, dyspnea), and possibly nausea related to hyperventilation [73]. The participants will also be strongly encouraged to immediately contact a member of the research team if they notice any adverse events. Study personnel will also inquire about chest discomfort or delayed-onset muscle soreness during IMT in weekly meetings to adjust IMT prescriptions and ensure tolerability of training intensity.
- Satisfaction
  Participation satisfaction and motivation to engage with the IMT program will be assessed in the intervention group using a standardized questionnaire administered at weeks 1, 4, and 8 after study enrollment.

#### Primary Pilot Outcome

The primary pilot outcome will be the change in MIP over the 8-week study period to demonstrate the efficacy of IMT, which has been shown to be an important physiological measure in several IMT studies [11,26,35,73].

#### Secondary Outcomes

The secondary outcome measures evaluated will include dyspnea, respiratory muscle structure and function, mood, and HRQL assessed at T_0_ and 8 weeks, as shown in Table 3.

**Table 3:** Study outcomes.

Outcome				Assessment measurement		Time point
**Primary outcome**						
	Respiratory muscle strength		MIP^a^ (cm H_2_O)		Baseline (T_0_) and 8 weeks (T_2_)	
**Secondary outcomes**						
	**Pulmonary function**					
		Spirometry and lung volumes	FEV1^b^ (L), FVC^c^ (L), TLC^d^ (L), RV/TLC^e^ (%)		Baseline (T_0_) and 8 weeks (T_2_)	
		Oscillometry	Oscillometry: respiratory impedance, reactance, and resistance		Baseline (T_0_) and 8 weeks (T_2_)	
		Respiratory muscle endurance time	Time (seconds) using the Philips Threshold trainer at 30% of baseline MIP		Baseline (T_0_), 4 weeks (T_1_), and 8 weeks (T_2_)	
		Diaphragm structure and function	Diaphragm thickness and thickening fraction		Baseline (T_0_) and 8 weeks (T_2_)	
	**PROMs^f^**					
		Dyspnea	MRC^g^ scale ratings, Borg dyspnea scale ratings, and Qualitative Dyspnea Scale ratings		Baseline (T_0_) and 8 weeks (T_2_)	
		Exercise and physical activity volume	Godin Leisure-Time Exercise Questionnaire scores		Baseline (T_0_) and 8 weeks (T_2_)	
		HRQL^h^	SF-36^i^ scores		Baseline (T_0_) and 8 weeks (T_2_)	
		Depression, anxiety, and stress	Depression, Anxiety, and Stress scale scores		Baseline (T_0_) and 8 weeks (T_2_)	
	**Cardiopulmonary exercise parameters**					
		Aerobic and anaerobic capacity	Peak aerobic capacity, anaerobic threshold, breathing reserve, and cycling power output		Baseline (T_0_) and 8 weeks (T_2_)	
		HR^j^ response and chronotropic response	HR recovery (HR at 1 minute after CPET^k^ – HR at the end of CPET) and chronotropic response index adjusted for age ([peak HR – resting HR × 100] / [220 – age] – [resting HR])		Baseline (T_0_) and 8 weeks (T_2_)	
	Neural activity during exercise (prefrontal cortex)		Oxyhemoglobin and total hemoglobin concentration changes		Baseline (T_0_) and 8 weeks (T_2_)	

^a^MIP: maximal inspiratory pressure.

^b^FEV1: forced expiratory volume in 1 second.

^c^FVC: forced vital capacity.

^d^TLC: total lung capacity.

^e^RV/TLC: residual volume/total lung capacity ratio.

^f^PROM: patient-reported outcome measure.

^g^MRC: Medical Research Council.

^h^HRQL: health-related quality of life.

^i^SF-36: Short Form-36.

^j^HR: heart rate.

^k^CPET: cardiopulmonary exercise test.

### Statistical Analysis

#### Proposed Data Analyses

For objective 1, we aim to compare respiratory muscle structure and function and PROMs between patients with hEDS or G-HSD and healthy controls and to evaluate the main correlates with dyspnea in patients with hEDS or G-HSD. Descriptive statistics will be used for continuous variables (mean and SD or median and IQR), and frequencies will be used for categorical variables. We will use multivariable regression analysis to compare any differences between groups across respiratory parameters and PROMs, adjusted for EDS subtype (hEDS or G-HSD). We will explore the associations with peak Borg dyspnea scores during CPET using multivariable regression, with possible determinants being MIP, diaphragm thickening fraction, PFC activity, and PFT parameters.

For objective 2, we aim to assess the feasibility of IMT and standard-of-care rehabilitation for improving respiratory muscle strength, exercise capacity, and PROMs compared with standard-of-care rehabilitation alone in patients with hEDS or G-HSD. Descriptive statistics will be used to summarize program adherence, recruitment, participant satisfaction, attrition, and safety in the hEDS or G-HSD study groups. Estimates of within- and between-group differences at T_0_, 4 weeks, and 8 weeks will be reported using means and 95% CI. Paired *t* tests and *t* tests will be used to compare the changes in the primary pilot outcome (MIP) and secondary measures over the 8-week study period within and between the 2 randomized groups.

#### Sample Size

##### Objective 1

With the prevalence of dyspnea (MRC score: 2 out of 5) in 50% of the patients with hEDS and G-HSD, we estimate that 75% of the patients with hEDS will report a Borg dyspnea score of ≥4 (moderate dyspnea) compared with 25% of healthy controls after cardiopulmonary exercise, as previously observed at our center [74]. Thus, with a sample size of 34 patients with hEDS or G-HSD and 17 healthy controls, we will have 90% power to observe a significant difference in moderate dyspnea (α level=.05). Furthermore, given an estimate of 10 participants for each covariate in multivariable regression modeling, we reckon that enrollment of 34 participants with hEDS or G-HSD will allow us to explore at least 3 determinants of dyspnea in our models.

##### Objective 2 (Primary Pilot Outcome)

Given an estimated MIP comparable with that of moderate COPD patients (mean 51, SD 15 cm H_2_O), we anticipate a mean increase of 10 (SD 3.5) cm H_2_O in MIP in the IMT and exercise training group compared with the hEDS or G-HSD usual care group (exercise training alone; 5 cm H_2_O) [75]. Assuming a maximum attrition rate of 20% among the 34 RCT participants with hEDS or G-HSD, we will have 90% power (α=.05) to observe a significant difference in MIP with 17 participants per group.

#### Data Management and Quality Assurance

Our research team is trained in the measurement protocols and has expertise in performing physical assessments and PROMs in individuals with respiratory conditions. The research team has critically appraised the peer-review comments from the 2020 GoodHope Ehlers-Danlos Syndrome foundation grant (Multimedia Appendix 1) and has incorporated them into the study protocol. Most of the study assessments will be conducted at the PFT laboratory of Toronto General Hospital by experienced respiratory technicians. All equipment (ultrasound, fNIRS, and oscillometry) has been applied in the laboratory setting previously. In addition, our research team has expertise in conducting web-based assessments in the participants’ home environment, including IMT, given the COVID-19 environment.

The research coordinator will guide the participants through each step of the study process. Standard operating procedures will be generated for each step related to data collection, data entry, and data handling. The research coordinator will ensure accuracy by periodically auditing the data in our research database. Although the research coordinator will attempt to have the participants complete the entirety of the assessments and questionnaires, if there are unanswered questions or missing data, the data will not be imputed, and analysis will be performed on the available data.

The current institutional and national research ethics guidelines will be followed to ensure data privacy, security, and protection. Participant data will only be accessible by the research study team, and participants will be assigned a random participant ID, which will be linked to all the data. All the study data will be securely stored on a password-protected server. In the event of inappropriate data breaches, the following actions will be undertaken: (1) prevent further breach of information and attempt to retrieve breached information and (2) notify the institutional research ethics board immediately. Further action might be taken according to the recommendations from the research ethics board. Modifications to the study protocol will be approved by the research ethics board before implementation, communicated to funding agencies, and outlined in future publications.

## Results

This research was funded in January 2021, and study enrollment began in August 2021 and will continue until December 2023. As of January 2023, a total of 4 participants with hEDS or G-HSD in the intervention group and 4 participants in the usual care group have completed the study, with 5 other participants with hEDS or G-HSD enrolled. A total of 11 healthy controls have completed the study. The results will be submitted for publication once data collection and assessments are complete.

## Discussion

### Summary

This study will help characterize the multifactorial nature of dyspnea experienced by individuals with hEDS and G-HSD. Given the known impairments in respiratory muscle strength in this population, this study will allow us to assess the synergistic effects of IMT and standard-of-care rehabilitation on dyspnea, PROMs, and exercise capacity, which have not been previously characterized. The findings of this study will inform the feasibility of a larger study using IMT and standard-of-care rehabilitation that will be appropriately powered to assess the effects of IMT on PROMs and clinical outcomes. Furthermore, this study has the potential to inform our understanding of the mechanistic effects that IMT may have on diaphragm structure and function, lung physiology, and neurocognitive adaptations in this population.

The literature describing respiratory manifestations in EDS and G-HSD has generally been limited to a few observational studies and case series involving mainly 4 subtypes: classical EDS, vascular EDS, hEDS, and G-HSD [2,9]. We chose to focus on the population with hEDS, as hEDS comprises the major subtype of EDS, and there is also a study highlighting respiratory muscle weakness and effectiveness of IMT [11]. The population with G-HSD was also chosen, as G-HSD is defined to be on the continuum of EDS and represents one of the most common diagnoses in our GEAR rehabilitation program, and the respiratory sequelae of G-HSD have not been well characterized [3]. Another important consideration factored into our selection of the EDS population was the risk of pneumothorax, which is generally lower in individuals with hEDS and G-HSD than in those with other subtypes. Thus, the inclusion of patients with hEDS and patients with G-HSD is owing to the representation of the majority (>90%) of the patients with EDS followed in our program.

We hypothesize that IMT and standard-of-care rehabilitation will provide a positive synergistic effect on respiratory muscle function, PROMs, and clinical outcomes. IMT has been shown to improve dyspnea by increasing respiratory muscle strength [76]. In addition, aerobic and strength training has been shown to increase the strength of the respiratory and limb muscles and improve oxygen use, ventilatory efficiency, and exercise capacity [20,31]. Furthermore, IMT can have positive effects on dyspnea through neurocognitive adaptations [77]. Breathing exercises may induce motor-sensory neural adaptations in the diaphragmatic and respiratory muscles, which can improve dyspnea tolerance [77]. It is also postulated that improvements in exercise capacity and dyspnea levels through IMT may result in greater independence in daily activities and improved HRQL [78]. Despite the proposed beneficial effects of IMT, the ideal duration remains to be determined, with 8 weeks chosen for this study based on the recommendation that a duration of at least 6 weeks is required to derive respiratory muscle adaptations [79-81].

Owing to the COVID-19 environment, a large proportion of research assessments and in-person care visits have transitioned to a web-based environment [82,83]. Patients often express concerns about the possibility of COVID-19 exposure and travel expenses for in-person assessments. Furthermore, the GEAR program has also adapted its format, with a proportion of web-based training sessions performed. Thus, we are closely monitoring enrollment numbers and if recruitment proves to be challenging because of the requirement for in-person visits, we will be able to amend the protocol to allow assessments to be performed via the web. In fact, most of the assessments in this study can be administered via the web, including all PROMs, respiratory muscle endurance testing, and IMT with electronic tracking on the device itself. The results of this study will demonstrate the potential of performing web-based IMT, especially with the emergence of tele-rehabilitation programs.

### Limitations

This study has several limitations that need to be highlighted. First, our study focuses on hEDS and G-HSD, which represent most EDS disorders in our program. However, the findings may not be generalizable across less prevalent EDS subtypes (eg, classic EDS or vascular EDS). Second, all participants in this RCT will be enrolled in the GEAR program, and we anticipate that any differences in training volumes or physical activity levels will be negligible between the groups after randomization, but any differences over the 8-week IMT program will be explored. Third, we are unable to monitor adherence to the GEAR program in the intervention group. Moreover, IMT may not be suitable for all participants because of time restrictions, training preferences, and work-school schedules. However, the web-based environment provides participants with the flexibility to uptake the IMT program that best suits their schedules.

### Conclusions

This study aims to provide a better understanding of the contributors to dyspnea in hEDS or G-HSD and highlight the association between dyspnea and respiratory muscle function, PROMs, and exercise capacity. The combination of IMT and standard-of-care rehabilitation training may provide a novel therapeutic strategy to improve respiratory muscle function and PROMs. A better understanding of the feasibility and efficacy of IMT and standard-of-care rehabilitation will provide insight into the use of IMT in the population with EDS and the population with G-HSD, given a shift toward tele-rehabilitation programs in the COVID-19 environment. This research will pave the way for future studies to evaluate respiratory management strategies in this population.

## Appendix

Multimedia Appendix 1 Peer review report from the 2020 GoodHope Ehlers-Danlos Syndrome Foundation grant competition.

## Abbreviations

CONSORT: Consolidated Standards of Reporting Trials
COPD: chronic obstructive pulmonary disease
CPET: cardiopulmonary exercise test
EDS: Ehlers-Danlos Syndrome
fNIRS: functional near-infrared spectroscopy
GEAR: GoodHope Exercise and Rehabilitation Program
G-HSD: generalized hypermobility spectrum disorder
hEDS: hypermobile Ehlers-Danlos Syndrome
HR: heart rate
HRQL: health-related quality of life
IMT: inspiratory muscle training
MIP: maximal inspiratory pressure
MRC: Medical Research Council
PFC: prefrontal cortex
PFT: pulmonary function test
PROM: patient-reported outcome measure
RCT: randomized controlled trial
T0: baseline

## Data Availability

The study data will be made available for review after trial completion and publication upon reasonable request from the corresponding author.
