# Supplementary material for: Evaluation and Management of Dyspnea in Hypermobile Ehlers-Danlos Syndrome and Generalized Hypermobility Spectrum Disorder: Protocol for a Pilot and Feasibility Randomized Controlled Trial
Source: JMIR Res Protoc. 2023 Mar 20;12:e44832. doi: 10.2196/44832 (PMC10131980; doi:10.2196/44832)
Supplement: Multimedia Appendix 1 [file resprot_v12i1e44832_app1.pdf]

December 22, 2020

Dr. Dmitry Rozenberg
Clinician Scientist
Toronto General Hospital Research Institute
200 Elizabeth Street, Room 13EN-229
Toronto, Ontario M5G 2C4

Dear Dr. Rozenberg,

Re: **Rozenberg, D; Mittal, N; McGillis, L; Lopez-Hernandez, L; Santa Mina, D; Reid, D; Goligher, E; Chow; C-W; Ryan, C; Kumbhare, D; Huszti; Champagne, K; Raj, SR; Evaluation and Management of Dyspnea in Hypermobile Ehlers-Danlos Syndrome**

2020 GoodHope Ehlers-Danlos Syndrome Foundation grant competition

I am pleased to advise you that your application to the 2020 GoodHope Ehlers-Danlos Syndrome Foundation grant competition has been approved for funding.

The reviewers noted the following:

- The application team includes a wide range of clinical and scientific expertise that is well suited to achieving the study outcomes
- You have proposed well thought out statistical analyses to determine predictors
- Consider study participant load and REB approval
- The scope of work proposed may be too broad

The reviewers noted the following:

- It is a good proposal and study overall but there appear to be some variation in the aims and measures. Aims state that HRQL will be evaluated (which then makes the use of the short form SF 36 a valid measure and can provide an composite mood score), however there is no aim stated to specifically measure anxiety and depression yet the HADS is included as a measure. This could add additional participant burden as they are being exposed to.

- If a mood score will suffice, the candidates should remove the use of the HADS and reduce the impact of the burden of study participation

- The plan to approach REB and initial thoughts on participant protection/considerations is not mentioned in the study write up (for example, participants suffering from dyspnea also likely have significant fatigue. Is it possible to achieve study purpose with less burdensome data collection strategies (e.g., use SF 36 physical and mental health composite scores versus using various measures to determine patient reported outcomes)

The Krembil Directorate Office will be in touch shortly to set up a fund for this award. If you require a change in the budget or the nature of expenses, please contact the office (krembil@uhnresearch.ca) for approval. We will be in touch at the one-year mark for a brief progress report.

If you have any questions or concerns relating to this award, please do not hesitate to contact the Krembil Directorate Office (krembil@uhnresearch.ca) or myself.

Please accept my best wishes for success in your research.

Yours sincerely,

Hance Clarke,
Staff Anesthesiologist
Director Pain Services
Director Good Hope Ehlers Danlos Clinic
Medical Director of The Pain Research Unit
Department of Anesthesia and Pain Management
Toronto General Hospital, University Health Network
Clinician Investigator, Krembil Research Institute
Co-Chair Knowledge Translation, University of Toronto Centre for the Study of Pain
Co-Lead, Centre for Cannabinoid Therapeutics, University Health Network

Below, please sign and return a copy of this letter to the Krembil Directorate Office (krembil@uhnresearch.ca) by January 15, 2021.

I have read and accept the terms of this award letter.

____

Dmitry Rozenberg

____

Date
